# Applying dental implant therapy in patients with oral lichen planus: a review of literature

**DOI:** 10.1186/s40729-020-00216-8

**Published:** 2020-05-27

**Authors:** Farnoosh Razmara, Mina Khayamzadeh, Ghazal Shabankare

**Affiliations:** 1 Craniomaxillofacial Research Center, Tehran University of Medical Sciences, Tehran, Iran; 2 Oral and Maxillofacial Surgery Department, School of Dentistry, Tehran University of Medical Sciences, Tehran, Iran; 3 Department of Oral and Maxillofacial Disease, School of Dentistry, Tehran University of Medical Sciences, International Campus, Tehran, Iran; 4 School of Dentistry, Tehran University of Medical Sciences, International Campus, Tehran, Iran

**Keywords:** Oral mucosa, Lichen planus, Oral lichen planus, Dental implants

## Abstract

Lichen planus (LP) is a violent, paranormal inflammatory disease that can affect the skin or any lining of the mucous membrane. LPs are a branch of immune-mediated inflammatory disease (IMID) that collaborates with the function and structure of the immune system that are precipitated through various etiological infectious agents. Oral lichen planus (OLP) is one of the most common kinds of IMID. These traumas might limit the normal life of patients and, in some cases, can be treated spontaneously. In patients who are affected by OLP, the dental clinicians must be capable of the proper diagnosis of the disorder. Dental implants are progressively applied for the treatment of partial or complete edentulism. Implant rehabilitation in OLP patients is one of the main challenges for patients and dental clinicians. There is not enough knowledge about this condition, and also medical documents are limited. In this study, by conducting a comprehensive review of literature, we tried to collect related data around the safety and success rate of implant rehabilitation in patients who suffer from OLP disorder. There proved to be no relation between implant survival rate and OLP diseases, but it is proven that some factors such as bone quality and fracture resistance, parafunctional habits, and resection of the marginal mandible could powerfully affect it. For evaluation of the advantages and disadvantages of applying implants in patients with OLP disorders, implementation of controlled studies is required.

## Introduction

Dental implants are progressively applied for the treatment of partial or complete edentulism. By referencing to the literature, it is proven that dental implants could remain safe and efficient in about 95% of cases after 10 years [1, 2], The convenience of patients in the selection of treatment method in dental implant therapy was successfully conducted for the patients with edentulism [3]. Oral mucosal disorders as the worst of the systemic diseases that involve the oral mucosa could make dental implant therapy more complicated. Relative and absolute contraindications for applying implant therapy contained a lot of localized and systemic diseases that have been proven to have less effect on implant efficiency period. Contraindication factors are such disorders like diabetes, bone disorders, osteoporosis, blood cancer (leukemia), functional disorders like gastrointestinal (GI) problems, inherited immunodeficiency disorders like immunosuppression, some systemic diseases, and also congenital disorders [4–10].

Favorable outcomes of applying an implant principally depend on the quality and quantity of the related factors of the bone. On the other hand, factors that affect soft tissues would have several effects on bone loss and implant efficiency. According to Jemt and Johansson’s [11] study on surgical implant treatment, the surrounding marginal bone around dental implants is frequently the primary bone loss area. On the other hand, the ability of the epithelial tissue to stick and seal this area is a very critical factor for an implant’s survival and efficiency. Hernandez et al. [12] have implemented a comprehensive study in terms of clinical outcomes of peri-implant peripheral giant cell granuloma and reported that various local conditions of mucosa may cause the fracture of the implants, while some researchers have assumed that the existence of epithelial diseases may be considered as local and biological contraindications for implant placement [5, 6].

Oral lichen planus (OLP) is a chronic mucosal inflammation and is frequently observed in clinical oral examinations. The main agent that is used for the treatment of symptomatic traumas of OLP is the corticosteroid that is applied instantly to the infected region [1, 13]. Lodi et al. [14] have discussed current controversies in oral lichen planus and reported that OLP disorders occur in almost 2.5% of individuals. The etiopathogenesis and further development of this disorder include multiple immune responses without antibody involvement which results in the damage of the epithelium and connective tissue.

The main characterization factors of OLP are recurrence and its related clinical modifications such as erosive, plaque-like, atrophic, bullous, reticular, and papular [15]. OLP disorder has frequently been reported in patients over 40 years, and as reported by Roopashree et al. [16], OLP has a female predilection. According to Gonzalez et al.’s study [17] in the algorithm of OLP treatment, planning, symptoms, the extent of body organs that are infected by OLP, patient medical history, and some extra factors should be considered carefully.

Whether to replace the missing teeth of OLP patients with dental implants or not is still the dilemma of oral and dental healthcare professionals, as it has been suggested that OLP can possibly give rise to implant failures due to the impaired adherence capacity of the epithelium to the implant surfaces. The fact that dental implants are becoming the popular prosthetic rehabilitation means it necessitates the existence of relevant clinical information on dental implant therapy for OLP patients. As possible drawbacks of dental implant placement for OLP patients is still a matter of conjecture, there is a clear need for further investigation of this issue [18]. Therefore, in this study, we aimed to evaluate the success rate of implant therapy in OLP patients through a comprehensive review of literature.

## Materials and methods

### Search strategy

Relevant published studies were searched for in EMBASE, MEDLINE, CDSR, PubMed, and CINAHL from 2000 to 2019 using the following keywords or, in case of PubMed database, medical subject headings: “oral mucosa”, “lichen planus”, “oral lichen planus”, “implant”, and “dental implants”. They were used alone or in combination using Boolean operators, OR and AND.

As inclusion criteria, any study on patients with OLP (before or after implant placement) receiving dental implant treatment was included. Full-text, English randomized controlled trails (RCTs), retrospective studies, prospective studies, cross-sectional, case series, and case reports involving human subjects were searched for. As exclusion criteria, articles describing extraoral lichen planus lesions, no dental implant placement, and patients with co-existing oral mucosal diseases were excluded.

In this article, we focused on answering the following question:

“Does OLP reduce the survival rate of dental implants?”

### Data extraction strategy

**Fig. 1:**
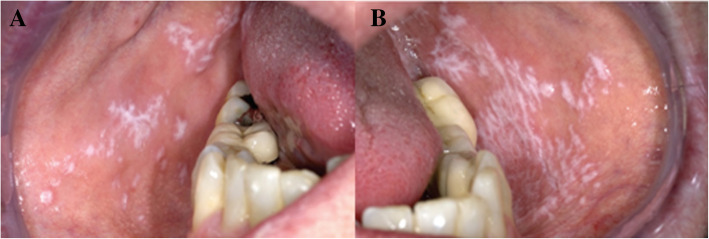
The selection process chart of articles related to current study based on the PRISMA method

All related information was extracted on specifications of the population of study, outcome criteria, and study interventions. Observational study type, the extent of studied articles, demographic information of patients, the type of OLP disease and its duration, number of placed implants, the rate of implant survival, and also the rehabilitation prosthetic were the criteria of the extracted data of the study. The schematic diagram shown in Fig. 1 clearly shows the methodology of the current study.

Heterogeneity of the selected studies regarding their differences in types of OLP, types of implants, types of studies, follow-up periods, and the onset of OLP (before/after implant placement) prevented valid mathematical combination of the collected data.

### OLP clinical aspects and symptoms

**Fig. 2:**
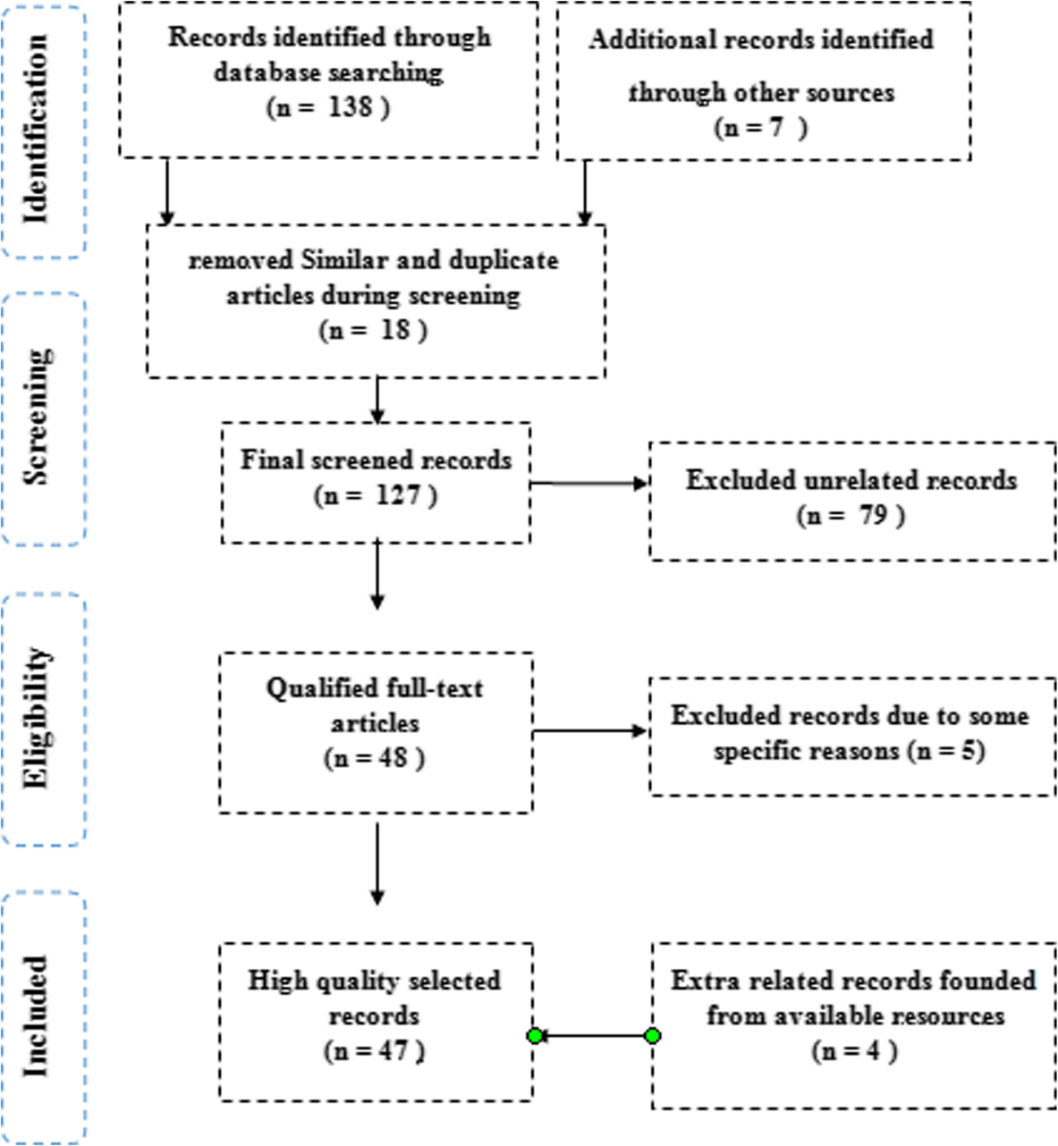
OLP typical presentation of an otherwise healthy patient (**a**, **b**). In such a condition, biopsy examination of tissue for medical diagnosis is required. Derived from Aghbari et al. [20]

The OLP course is specified by its healing progress and recurrence time, so that the symptoms and signs could last for weeks or months. The OLP symptoms may differ from feeling of roughness to pain or itching in the mucosa of the mouth specially at the time of eating spicy foods [19]. As reported by Salgado et al. [19], when the gingival tissues get involved, the main complaint may be bleeding at the time of brushing. OLP disorder may appear with different clinical illustrations like annular, erythematous, bullous, reticular, ulcerative, plaque, and papular. Various clinical symptoms may occur together or might be changed during a period of time. The most frequent affected sections of the mouth are gingiva, the oral mucosa, and the tongue. In most patients, the recognition process of OLP could be done clinically, especially when it is presented in the clinical reticular form (Fig. 2). On the other hand, when it is difficult to distinguish various types of OLP, different subordinate types of leukoplakia, lichen planus disorders, chronic ulcerative stomatitis, pemphigoid autoimmune disorder of the mucous membrane, and even the various stages of syphilis, medical biopsy test and immunohistochemical (IHC) examination would be effective for examination of tissue histopathology [20].

### OLP dental management

The clinical diagnosis and management of OLP disorder, with its broad range of inflammatory traumas that would affect the mucous membrane lining inside of the mouth, is a serious challenge for dentists. For achieving essential knowledge about OLP disorder and providing extra suggestions about clinical dental management of OLP specially implant therapy methods, some related discussions based on literature and on the long periodic clinical experiences are reviewed in this study.

### OLP epidemiology

Oral lichenoid disorder which is known as a precancerous condition is required to be meticulously recognized and treated as a separate disorder from OLP variations [21]. Cellular immune responses have an important role that could be applied antagonistically towards special connective tissue in regressive patterns. Initial diagnostic alongside confirmatory tests could be carried out by medical procedure like biopsy, immunofluorescence (IF) laboratory techniques, immunoprecipitation (IP) techniques, and identifying techniques of immunoblotting [22]. Initial adequate recognition of the disease is a very critical step for planning an effective treatment method and increasing survival rate of patients. Oral lichen planus comes in various clinical forms and could be misdiagnosed. This status occurs because of some various systemic causes like diabetes mellitus (DM) and hypertension (HTN or HT) that are also known as high blood pressure (HBP). Similar disorders have been reported in the nails, esophagus, trunk, larynx, cutaneous innervation, and scalp [23].

OLP disease is more common among people with mean age of 45 years and has a female predilection [15]. As reported by Pandhi et al. [24], this disorder is infrequent among children. Payeras et al. [25] have come to the conclusion that by focusing on the etiopathogenesis of lichen planus, LP disorder is mainly the consequence of responses of the T-cells of the immune system, while the actual etiopathogenesis and subsequent development of LP is not well known yet. Chen et al. [26] have done an experimental study on the triggers of oral lichen planus flares and the potential role of trigger avoidance in disease management and claimed that psychological factors could play a significant role in LP disease. By reviewing the course of the oral lichen planus, Carbone et al. [27] evaluated the association between Hepatitis C (hepatitis C virus) and lichen planus and concluded that there is a considerable unknown dependency among them. By investigating about clinical characteristics of patients with concomitant oral lichen planus and thyroid disease, Robledo et al. [28] reported that there is an association among thyroid diseases and patients who are affected by the LP disease. Additionally, Tovaru et al. [29] investigated the association of gallbladder disease with OLP and estimated that approximately 20% of OLP patients suffer from gallbladder disease, too. However, the OLP patients must be frequently checked for possible presence of the aforementioned disorders.

Hirota et al. [30] have investigated the possible association between oral lichen planus and drug intake and concluded that OLP traumas that are alike lichenoid reactions and/or lichenoid lesions may sometimes be produced due to the application of the systemic remedies. Rice and Hamburger [31] have worked on drugs possibly causing lichenoid lesions and introduced some of the drugs that may cause lichenoid lesions that are mentioned in Table 1. On the other hand, they reported that direct elongated contact with liquid mercury amalgam filling or other medical materials used in dentistry such as glass ionomer cement and dental composites might agitate a lichenoid trauma in the mucosa of the mouth. Moreover, oral traumas of patients with persistent graft-versus-host disease (GvHD) that is accompanied by homogenous stem cell transplantation may clinically mimic OLP lesions.

**Table 1:** Several remedies that could cause lichenoid traumas. Derived in according to Rice and Hamburger [31]

As an example	Drug class
Captopril	Antihypertensives
Tolbutamide	Oral hypoglycemics
Ibuprofen	Non-steroidal anti-inflammatory drugs
Gold, penicillamine	Second line anti-arthritics
Allopurinol	Oxidase inhibitors
Lorazepam	Psychoactive drugs
Furosemide	Diuretics
Cloroquine	Antiparasitic
Tetracycline	Antimicrobial agents, including mouth rinses
Iodides, quinidine	Miscellaneous drugs

### Implant therapy

According to Pentenero et al. [32], who conducted studies on the prevalence of oral mucosal lesions in adults, injuries created via artificial body parts may obstruct the recuperation of traumas induced by the pharmacological OLP treatment. Additionally, as cited by Kivovics et al. [33], oral rehabilitation supported by implant therapy might reduce the connection of affected mucosae of the mouth with materials of the artificial body part (implant), and on the other hand, it fixates the artificial body part and reduces the frictional force among the dentures and the oral mucosa. Emami et al. [34] claimed that application of dental implants in patients with OLP in the past was not suggested because of the possibility of painful inflammation traumas like oral mucositis before implant therapy that may cause the failure of implant therapy. Additionally, some dendritic cells like Langerhans and epidermal cells like keratinocytes in OLP traumas could increase the secretion of some pro-inflammatory components [34].

Taubman and Kawai [35] have investigated the involvement of T-lymphocytes in periodontal disease in the direct and indirect induction of bone resorption and cited that significant small proteins that have a critical role in cell signaling (cytokines) are detected to have an important role in the local resorption of the bone and also might damage the alveolar bone where implants are placed.

According to the reviewed literature, up to now, no particular comprehensive guidance on the advantages and disadvantages of applying implants in patients who are affected by OLP exists. As cited by Candel et al. [36] who have worked on dental implants in patients with oral mucosal alterations, the information on applying implant therapy in patients with OLP disease is limited. Anyway, a comprehensive review of literature on the advantages and disadvantages of rehabilitation of oral implants in patients with OLP disease should be implemented.

Scully et al. [37] have studied dental endosseous implants in the medically compromised patients and claimed that dental implants are progressively applied to treat edentulous patients, and this type of oral rehabilitation has more successful outcomes compared to other methods. According to their report, in some patients with systemic disorders, the application of dental implants is not performed. As they mentioned, systemic diseases could affect the tissues of the mouth, enhancing the risk of other pathologies and also the obstruction of the patient’s treatment process. Additionally, according to Bornstein et al.’s [38] reports, the management of the mentioned diseases with drugs or extra therapies could influence dental implants or the supporting soft tissue. Sometimes mucosal traumas of the mouth and systemic diseases are considered as contraindications for applying implant therapy. Despite the advantages of this kind of treatment, it can increase the risk factors of traumas at the same time.

Reichart at al [39]. conducted a comprehensive study about the effect of OLP disorders on dental implants and claimed that both the quality of the alveolar bone and oral mucosa are determining factors for applying implant therapy. Esposito et al. [40] investigated the implant-retained overdentures in two patients with severe lichen planus and finally suggested that in patients who were affected by OLP disease, the volume of the epithelial tissues for immediate adherence of the implant titanium surface is modified. Cortes et al. [41] have worked on oral lichenoid disease as a premalignant condition and reported that among five patients who received implant therapy, the success rate was approximately 100%. According to Esposito et al. [40], the application of dental implants instead of overdentures reduces the occurrence of erosive traumas, enhances the comfort of patient, and also increases the oral function. Cortes et al. [41] stated that OLP is a precancerous disorder and its rate of malignant transformation is under 1%. However, the possibility of malignant transformations should be considered carefully. According to the reviewed literature, rehabilitation of OLP patients who have received implant therapy is about 100%. So, for achieving adequate advantages of applying implant in such patients, periodic examination of patients is required.

## Results

According to collected records, among the large number of medical publications that considered OLP disease, those that were more related to the criteria of the study subject are shown in Table 2. Most of the studied records were clinical single case reports with factual description of the features [13, 39, 44, 47]. Additionally, some retrospective, prospective, and also case–control studies were investigated [10, 43, 46]. Moreover, both female and male patients were considered in the studies. Mostly mature patients were detected to be affected by erosive OLP and some of them were diagnosed with reticular OLP and a few patients were detected with no types of OLP. About three publications have discussed the OLP duration and clinical signs and symptoms [39, 44, 47]. The collected information about dental implant systems, applicated denture types, the rate of implant survival, and the period of time that patients followed-up are mentioned in Table 2. About nine studies reported successful implant therapy in patients with OLP. Only few studies mentioned the types of applied implants and, they reported that, just 2 implants had flat machined surface, where some other reported application of implants with micro rough surfaces.

**Table 2:** The collected information around type of the studies, dental implant systems, applied denture type, the rate of implant survival, and the period of time that patients were followed-up

Authors	Type of study	Number of applied implants	Implant systems and features	Applied denture type	Survival rate of the implants (%)	Follow-up period (months)
Esposito et al. [42]	Case report	4	Straumann TL and micro rough surface	Overdenture	100	21
Oczakir et al. [3]	Retrospective study	4	Straumann TL and micro rough surface	Fixed complete denture	100	72
Reichart [39]	Case report	10	HaTi [2], Camlog [1], micro rough surface ZL-Duraplant [1], micro rough and anodically oxidized surface [6]	Fixed partial prostheses	100	156
Czerninski et al. [43]	Retrospective study	3	NA	Fixed partial prosthesis	0	36
Gallego et al. [44]	Case report	2	NA	Overdenture	0	36
Hernandez et al. [10]	Prospective study	56	Nobel Biocare, micro rough and anodically oxidized surface	Fixed partial prostheses	100	53.5
Diz et al. [43, 45]	Retrospective study	54	NA	NA	100	63
Lopez-Jornet et al. [46]	Cross-sectional study	56	NA	3 Overdentures 13 fixed partial prostheses	Implant survival rate: NA peri-implant mucositis: 10 peri-implantitis: 14 bone loss: 10 mobility: 2	42
Total		191			0–100% weighed mean 95.3%	Weighed mean 53.8 SD ± 18.3

## Discussion

According to the reviewed literature, the majority of oral mucosal disorders were reported in patients with OLP. By studying implant treatment in patients with oral lichen planus, Hernandez et al. [10] conducted the first prospective study that contained about 20 patients with OLP. In their study, implant insertion during erosive OLP phases was avoided. Moreover, after inserting implants and during follow-up examinations, ulcerations and erosions were cured with Clobetasol propionate, and also patients were instructed to use Clobetasol dipropionate three times a day for avoiding remissions. Finally, the authors concluded that in OLP patients who received dental implant therapy, such wounds that are impaired are not the same as in patients who do not have any disorders of the mucous membrane of the mouth.

By conducting a retrospective study in term of OLP disease and dental implants, Czerninski et al. [43] compared clinical symptoms in patients who were affected by OLP and were treated by implant therapy or not, but they did not report any statistical difference between them. According to their results, OLP disease could not be considered as a contraindication for dental implant application. Pons and Jornet [18] conducted a narrative review on dental implants in patients with OLP and cited that in spite of the fact that the number of OLP patients who were treated with implant therapy is low, it might be concluded that the failure of implant therapy in some patients is not because of OLP disorder but due to some other factors like the poor quality of the underlying bone. Therefore, they suggested that before applying implant therapy, the dental specialists must consider disadvantages against advantages and also complications of the applied method in addition to the remedy costs.

According to the reviewed articles, it could be concluded that further studies are required for clarifying the survival rate of applied implant therapy in OLP patients. As cited by some researchers [10, 43], implant therapy must be applied in stages that OLP disorder is remedied. By implementing some comprehensive research on dental implants in the medically compromised patients, Diz et al. [45] mentioned that precise hygiene of the oral mucosa and frequent follow-up sessions are important factors that could have a great influence on implant survival rate in patients with OLP. In a precise report in terms of histopathologic observations on late oral implant failures, Esposito et al. [40] claimed that parafunctional habits of OLP disorder as well as the poor quality of the bone were the main factors of implant failure. In a clinical report on implant-retained overdentures for two patients with severe lichen planus, Esposito et al. [42] reported that 2 implants were placed in the lower canine area and the overdentures were held by O-ring overdenture attachments. In a comprehensive study on oral lichen planus and dental implants, Reichart [39] reported that implant therapy was applied without any complications in 3 patients with OLP.

## Conclusion

This comprehensive study concentrated on the outcomes of implant therapy in patients with OLP. According to the achieved results, it could be concluded that the survival rate of implants was similar to the patients with healthy mucous membrane of the mouth. However, advanced clinical examinations through respective studies containing control groups with patients who are not affected by OLP disease are essential for primary conclusions. Due to the fact that the number of publications concerning this issue is few, no reliable guidelines are available as a comprehensive treatment method for this disorder. According to the present study, there is no contraindication for applying implant therapy in patients who are affected by OLP disorders. At the same time, most of the authors emphasized that implant survival periods should be compared with patients who are not affected by OLP disorders. Finally, it could be concluded that, for more appropriate outcomes, adequate treatment guidelines should be conducted for OLP patients in terms of dental implant application.

## Data Availability

The datasets used and/or analyzed during the current study are available from the corresponding author on reasonable request.
